# Virus-induced gene silencing is a versatile tool for unraveling the functional relevance of multiple abiotic-stress-responsive genes in crop plants

**DOI:** 10.3389/fpls.2014.00323

**Published:** 2014-07-08

**Authors:** Venkategowda Ramegowda, Kirankumar S. Mysore, Muthappa Senthil-Kumar

**Affiliations:** ^1^Department of Crop Physiology, University of Agricultural Sciences, GKVKBangalore, India; ^2^Plant Biology Division, The Samuel Roberts Noble FoundationArdmore, OK, USA; ^3^National Institute of Plant Genome ResearchNew Delhi, India

**Keywords:** abiotic stress, functional genomics of crop plants, plant viruses, post-transcriptional gene silencing, virus-induced gene silencing

## Abstract

Virus-induced gene silencing (VIGS) is an effective tool for gene function analysis in plants. Over the last decade, VIGS has been successfully used as both a forward and reverse genetics technique for gene function analysis in various model plants, as well as crop plants. With the increased identification of differentially expressed genes under various abiotic stresses through high-throughput transcript profiling, the application of VIGS is expected to be important in the future for functional characterization of a large number of genes. In the recent past, VIGS was proven to be an elegant tool for functional characterization of genes associated with abiotic stress responses. In this review, we provide an overview of how VIGS is used in different crop species to characterize genes associated with drought-, salt-, oxidative- and nutrient-deficiency-stresses. We describe the examples from studies where abiotic stress related genes are characterized using VIGS. In addition, we describe the major advantages of VIGS over other currently available functional genomics tools. We also summarize the recent improvements, limitations and future prospects of using VIGS as a tool for studying plant responses to abiotic stresses.

## Introduction

The recent advances in next-generation sequencing technology has enabled sequencing of stress-specific transcriptomes and genomes of stress tolerant and susceptible cultivars (Morozova and Marra, 2008). Furthermore, an inventory of genes showing altered expression under several abiotic stresses has been established for many crop species by expressed sequence tag (EST) analysis (Gorantla et al., 2007; Wani et al., 2010; Blair et al., 2011). In contrast to the enormous progress made in generating sequence information, functional analysis of genes is lagging behind. Although *in silico* approaches and comparative genomic strategies have provided initial clues about the identity and function of abiotic-stress-responsive genes in many crop species (Gorantla et al., 2007; Tran and Mochida, 2010; Soares-Cavalcanti et al., 2012), comprehensive functional characterization tools are necessary for understanding the precise role of these genes in combating abiotic stresses. Mutant plants generated by chemical mutagenesis (Saleki et al., 1993), T-DNA tagging (Koiwa et al., 2006), and transposon tagging (Zhu et al., 2007) have been used for understanding stress tolerance. However, the generation of large-scale mutant populations requires tedious and laborious efforts, and identification of mutated genes is a lengthy process. RNAi is another tool used for studying the functional relevance of various abiotic-stress-related genes (Guo et al., 2002; Senthil-Kumar and Udayakumar, 2010), but this requires time-consuming genetic transformation. Therefore, in order to quickly study the function of a large number of genes identified through abiotic-stress-specific transcriptome profiles in several crop species and their wild relatives, alternative high-throughput tools are needed. Virus-induced gene silencing (VIGS) has emerged as a successful gene knockdown technique in several crop species in part because it does not require transformation (Baulcombe, 1999; Burch-Smith et al., 2004; Senthil-Kumar and Mysore, 2011a) (Supplementary Table 1). Over the past several years, VIGS has been successfully used to understand the abiotic stress tolerance mechanisms in crop plants (Senthil-Kumar and Udayakumar, 2006; Senthil-Kumar et al., 2008; Manmathan et al., 2013). In this review, we discuss the utility of this powerful technique to study genes involved in abiotic stress tolerance. We also discuss the mechanism of VIGS and list the VIGS vectors available for a wide range of crops and novel ways for application of VIGS to carry out functional analysis of abiotic-stress-responsive genes. Further, the recent improvements in VIGS protocol, limitations and future prospects are discussed.

## Mechanism of VIGS and genesis of VIGS vectors

VIGS is a post-transcriptional gene silencing (PTGS)-based technique (Baulcombe, 1999), and it exploits the natural defense mechanisms employed by plants to protect against invading viruses (Voinnet, 2001). Plants infected by viruses induce double stranded RNA (dsRNA) mediated PTGS which degrades viral RNAs. For VIGS, the viral genomes are modified by removing genes which induce virus symptoms and cloning the cDNAs of viral genomes into binary vectors under CaMV35S promoter along with convenient multiple cloning sites to facilitate insertion of target gene fragments (Voinnet, 2001; Liu et al., 2002a,b). Viruses that do not have suppressors of gene silencing or have only weak suppressors are modified as VIGS vectors to induce PTGS-mediated degradation of target plant mRNAs (Li and Ding, 2001; Cao et al., 2005). VIGS vectors are constructed by cloning a fragment (usually 300–500-bp) of the plant target gene with efficient siRNA generation and no off-target genes into the modified viral genome (http://bioinfo2.noble.org/RNAiScan.htm) (Xu et al., 2006). The recombinant virus is then introduced into plant cells through *Agrobacterium tumefaciens*-mediated transient expression or *in vitro* transcribed RNA inoculation or direct DNA inoculation (Supplementary Table 2). After the recombinant virus is introduced into plant cells, the transgene is amplified along with the viral RNA by either an endogenous or a viral RNA-dependent RNA polymerase (RdRp) enzyme generating dsRNA molecules (Dalmay et al., 2000; Mourrain et al., 2000). These dsRNA intermediates are then recognized by DICER-like enzymes which cleave dsRNA into small interfering RNAs (siRNAs) of 21- to 25-nucleotides (Deleris et al., 2006). The double stranded siRNAs are then recognized by the RISC complex. The RISC complex uses the single stranded siRNAs and identifies complementary RNA sequences in the cell and degrades them (Fagard et al., 2000; Morel et al., 2002) (Supplementary Figure 1). VIGS has been shown to occur for a shorter period of approximately 3 weeks and the efficiency decreases after a month resulting in partial or complete recovery of plants from the silencing (Ratcliff et al., 2001; Hiriart et al., 2003; Ryu et al., 2004) (Supplementary Figure 2A). However, recent evidences suggest that some VIGS vectors can be used to maintain the gene silencing for several months by suitably modifying plant growth conditions that favor viral multiplication (Fu et al., 2006; Tuttle et al., 2008; Senthil-Kumar and Mysore, 2011b, 2014) (Supplementary Figure 2B) and can transmit to next generation (Senthil-Kumar and Mysore, 2011b) behaving like stable transgenic plants (Supplementary Figure 2C).

To date, about 35 DNA or RNA viruses have been modified as VIGS vectors (Senthil-Kumar and Mysore, 2011a). The VIGS vector resources available for crop plants are listed in Supplementary Table 1. Interestingly, the ability of certain viruses to infect a large number of host plants enabled the use of a single VIGS vector for gene silencing in several plant species (Robertson, 2004). For example, *Tobacco rattle virus* (TRV)-based VIGS vector is one of the most widely used VIGS vectors due to its ability to infect a wide range of host plants, systemic spread throughout the host plant including meristem, and lack of severe virus-associated symptoms in the infected plant (Valentine et al., 2004; Martín-Hernández and Baulcombe, 2008). TRV is a positive single stranded RNA virus with bipartite genome (RNA1 and RNA2). The RNA1 contains genes encoding RNA-dependent RNA polymerase, movement protein and 16K cysteine rich protein (Macfarlane, 1999). The RNA2 contains gene encoding coat protein and restriction sites for cloning the gene of interest (Liu et al., 2002b). Successful TRV-based VIGS requires infiltration of both RNA1 and RNA2 components. The TRV-based vector has been successfully demonstrated in functional analysis of abiotic-stress-responsive genes in model plants like *Nicotiana benthamiana* (Senthil-Kumar et al., 2007) and crop plants like tomato (*Solanum lycopersicum* and *S. pimpinellifolium*) (Senthil-Kumar and Udayakumar, 2006; Li et al., 2013; Virk et al., 2013), chili pepper (*Capsicum annuum*) (Lee et al., 2010; Choi and Hwang, 2012; Lim and Lee, 2014) and rose (*Rosa hybrid*) (Dai et al., 2012; Liu et al., 2013; Jiang et al., 2014).

Another source of VIGS vectors used for silencing of abiotic stress genes are the novel two-component system based on satellite-viruses along with helper viruses. In nature satellite-viruses are totally dependent on other viruses for replication (Tao and Zhou, 2004; Cai et al., 2007). An example of the DNA virus based two-component system is a satellite-virus-based vector, DNAβ, which was used along with *Tomato yellow leaf curl china virus* (TYLCCNV) as a helper virus to study the genes involved in abiotic stress responses in tomato (He et al., 2008; Guo et al., 2010). *DNAβ satellite virus* is devoid of the undesired effects of virus infection and instead functions to deliver the target gene fragment. RNA virus based VIGS systems with satellite and helper RNAs have also been developed. Here the satellite virus vector helps to deliver RNA into plants and the helper viruses supply replication and movement proteins. The advantage of two-component system is, it produces stronger silencing phenotypes compared to the satellite viruses alone (Gosselé et al., 2002).

In contrast to dicotyledonous plants, monocotyledonous plants have only a few VIGS vectors to date (Scofield and Nelson, 2009; Hema et al., 2013). Among these, the *Barley stripe mosaic virus* (BSMV)-based vector is the most widely used VIGS vector for functional analysis of abiotic stress genes in wheat (*Triticum aestivum*) (Kuzuoglu-Ozturk et al., 2012; Kang et al., 2013; Manmathan et al., 2013) and barley (*Hordeum vulgare*) (Liang et al., 2012). The availability of other vector resources and the potential of VIGS in monocotyledonous species have been comprehensively reviewed recently (Scofield and Nelson, 2009; Hema et al., 2013).

## Recent improvements in VIGS

Apart from a number of new VIGS vectors developed to suit a wide range of crop species, existing VIGS vectors and the technique have undergone several improvements in the recent past. For example, viral vectors have been modified to improve silencing efficiency. Recently, the RNA1 component of the bipartite TRV-vector was modified to serve as a VIGS vector which can infect plants systemically in the absence of RNA2 (Deng et al., 2013). This vector was developed by partially removing the 16K cysteine rich protein. The advantage of 16K protein removal is that it creates space for target gene cloning which otherwise cloned in RNA2 and also reduces the silencing suppression capacity of TRV. Furthermore, attempts have been made to identify gene-silenced tissues through a VIGS vector. For example, a *GREEN FLUORESCENT PROTEIN* (*GFP*) gene has been tagged to the coat protein gene of *TRV2* for easy identification of silenced tissue (Tian et al., 2014). This will help in tracing only green fluorescent tissues that have the virus, which are expected to have silencing, and hence facilitate the use of these tissues for abiotic stress assays. Some VIGS vectors have also been used to induce transcriptional gene silencing (TGS). Cloning of endogenous target gene promoter into viral vector and delivery into plants results in the production of siRNAs homologous to the endogenous gene promoter. These siRNAs facilitate RNA-directed DNA methylation (RdDM) and histone modifications, resulting in RNA-mediated gene silencing (Kanazawa et al., 2011). This can help suppress the regulators of abiotic stress response. In addition to improvements in VIGS vectors, VIGS procedure has been modified to perform silencing in different tissues. Gene silencing has been demonstrated in detached plant parts like petals (Dai et al., 2012), leaves and fruits (Romero et al., 2011; Ramegowda et al., 2013). This will facilitate high-throughput silencing and multiple stress impositions. VIGS has also been used to silence genes during tissue culture and callus development (Anand et al., 2007) which can facilitate precise stress imposition and high-throughput screening.

## VIGS for studying abiotic stress responses in crop species

VIGS has been used to investigate gene functions under abiotic stresses in model species. These studies involving model plants (Ahn et al., 2006; Moeder et al., 2007; Qian et al., 2007; Senthil-Kumar et al., 2007; Ahn and Pai, 2008; Cho et al., 2008; Hong et al., 2008; Sarowar et al., 2008; Govind et al., 2009; Ré et al., 2011) are not discussed in this review; instead, the main focus is given to studies involving crop plants. Recently, development of a wide range of VIGS vectors with high silencing efficiency has expanded the application of VIGS to several crop species for studying abiotic-stress-responsive genes (Table 1). The following sections enumerate the studies in which VIGS was used to characterize abiotic-stress-responsive genes in crop plants.

**Table 1:** **List of abiotic-stress-related genes silenced in crop plants using VIGS**.

**VIGS vector**	**Crop species**	**Silenced target gene**	**Abiotic stress**	**Changes in gene-silenced plants exposed to stress (compared to vector control plants)**	**References**
BSMV	Wheat	*TaEra1* (*ENHANCED RESPONSE TO ABSCISIC ACID*), *TaSal1* (*INOSITOL POLYPHOSPHATE 1-PHOSPHATASE*)	Drought	Increased relative water content (RWC), increased water use efficiency (WUE), reduced stomatal conductance, reduced transpiration rate and higher plant vigor	Manmathan et al., 2013
		*TaBTF3* (*BASIC TRANSCRIPTION FACTOR 3)*	Drought	Wilted and curled leaves under severe drought, higher water loss rate (WLR), decreased RWC and survival rate, lower free proline content, and increased membrane leakage	Kang et al., 2013
		*TaPGR5 (PROTON GRADIENT REGULATION 5)*	High light-induced photo-inhibition	Inhibition of photosynthesis, reduced non-photochemical quenching, increased membrane damage, anthocyanin and malondialdehyde (MDA) accumulation	Yuan-Ge et al., 2014
	Wild emmer wheat	*TdAtg8 (AUTOPHAGY-RELATED 8)*	Drought	Decreased chlorophyll content and increased MDA	Kuzuoglu-Ozturk et al., 2012
	Barley	*HvHVA1* (*H. VULGARIS ABUNDANT PROTEIN*)	Drought	Higher WLR in detached leaves, less survival, and retarded growth with reduced height and less total dry weight	Liang et al., 2012
		*HvDhn6* (*DEHYDRIN*)	Drought	Less survival, retarded growth and reduced total dry weight	Liang et al., 2012
BPMV	Soybean	*GmRPA3* (*REPLICATION PROTEIN A*)	Iron deficiency	Reduced chlorosis, increased chlorophyll, stunting and shorter internode	Atwood et al., 2014
PEBV	Pea	*PsSym19* (*SYMBIOTIC*)	Arbuscular- mycorrhizal- symbiosis-associated Pi uptake	Less development of arbuscules and vesicles in the root cortex of silenced plants	Grønlund et al., 2010Gr
		*PsPT4* (*PUTATIVE PI TRANSPORTER*)	Arbuscular-mycorrhizal-symbiosis-associated Pi uptake	Reduced phosphate uptake in new roots	Grønlund et al., 2010
		*TRX-F, TRX-M* (*THIOREDOXIN*)	Oxidative stress	Pale-green phenotype, reduction in the following: Mg chelatase activity, 5-aminolevulinic acid synthesis, chlorophyll, carotenoid pigment, photosynthesis and expression of tetrapyrrole biosynthesis pathway genes and increased accumulation of ROS	Luo et al., 2012
TRV	Tomato	*Sllea4* (*LATE EMBRYOGENESIS ABUNDANT PROTEIN 4*)	Drought or oxidative stress	Leaf wilting, reduced osmotic adjustment and cell viability, accumulation of higher superoxide radicals	Senthil-Kumar and Udayakumar, 2006
		*SpMPK1* (*MITOGEN-ACTIVATED PROTEIN KINASE 1*), *SpMPK2 (MITOGEN-ACTIVATED PROTEIN KINASE 2)*, *SpMPK3* (*MITOGEN-ACTIVATED PROTEIN KINASE 3*)	Drought or ABA or oxidative stress	Reduced survival, higher water loss in detached leaves, increased stomatal closure in response to ABA and increased H_2_O_2_ production in presence of ABA	Li et al., 2013
		*SlMPK4* (*MITOGEN-ACTIVATED PROTEIN KINASE 4*)	Drought	Early leaf wilting	Virk et al., 2013
	Chili pepper	*CaPO2* (*PEROXIDASE 2*)	Salt or osmotic stress	Reduced chlorophyll content and increased lipid peroxidation	Choi and Hwang, 2012
		*CaRAV1* (*RELATED TO ABI3/VP1*), *CaOXR1* (*OXIDOREDUCTASE 1*)	Salt or osmotic stress	Severe bleaching of leaf discs, loss of chlorophyll and increased lipid peroxidation	Lee et al., 2010
		*CaMLO2* (*MILDEW RESISTANCE LOCUS O*)	Drought	Less water loss and lipid peroxidation	Lim and Lee, 2014
	Rose	*RhNAC2* (*NAC TRANSCRIPTION FACTOR 2*), *RhEXPA4* (*A-TYPE EXPANSIN 4*)	Dehydration	Reduced fresh weight, petal width and recovery from dehydration	Dai et al., 2012
		*RhNAC3* (*NAC TRANSCRIPTION FACTOR 3*)	Dehydration	Reduced cell expansion during recovery	Jiang et al., 2014
		*RhACS1* (*ACC SYNTHASE 1*), *RhACS2* (*ACC SYNTHASE 2*)	Dehydration	Reduced ethylene production and cell density decreased	Liu et al., 2013
		*RhETR3* (*ETHYLENE RECEPTOR*)	Dehydration	Inhibition of petal expansion and cell expansion	Liu et al., 2013
TYLCCNV	Tomato	*SlGRX1* (*GLUTAREDOXIN 1*)	Oxidative or drought or salt stress	Reduced chlorophyll, leaf wilting, curled leaves and reduced RWC under drought; no further growth with wilted leaves and reduced chlorophyll under salt stress	Guo et al., 2010
		*SlFRO1* (*FERRIC CHELATE REDUCTASE 1*)	Nutrient deficiency	Reduced ferric chelate reductase activity in roots	He et al., 2008

### Drought stress tolerance

VIGS is a valuable tool for functional validation of drought-responsive genes identified from transcript profiling of plants exposed to drought stress. TRV-VIGS-mediated silencing of *lea4*, a gene encoding late embryogenesis abundant protein (LEA), resulted in increased susceptibility of tomato plants to drought stress. This gene was identified from a subtracted cDNA library for drought-stress-responsive genes (Gopalakrishna et al., 2001). At a given drought stress level, *lea4*-silenced plants wilted faster and recovered slower upon re-watering than the wild-type and vector control plants. *lea4*-silenced plants also exhibited reduced osmotic adjustment, reduced cell viability and higher superoxide radical levels (Senthil-Kumar and Udayakumar, 2006). In another study, a *GLUTAREDOXIN* gene, *SlGRX1*, was shown to regulate the drought stress response in tomato using a satellite-virus-based vector, DNAmβ (Guo et al., 2010). Under drought stress, silenced plants showed decreased chlorophyll content and decreased relative water content (RWC) compared to vector control plants (Guo et al., 2010). To study the role of mitogen-activated protein kinases (MAPKs) in drought tolerance of *S. pimpinellifolium*, a wild species of tomato, *SpMPK1, SpMPK2*, and *SpMPK3* genes were silenced individually or together using TRV-VIGS. Results suggested that co-silencing of *SpMPK1/SpMPK2* impaired ABA- and H_2_O_2_-induced stomatal closure and enhanced ABA-induced H_2_O_2_ production. But this response was not seen when *SpMPK1* and *SpMPK2* were silenced individually, suggesting these two genes are functionally redundant. This indicates that VIGS can be used to study functionally redundant genes. Reduced drought tolerance was also seen in *SpMPK3* alone and *SpMPK1*/*SpMPK2*/*SpMPK3* co-silenced plants (Li et al., 2013). Similarly, silencing of the *SlMPK4* gene in tomato resulted in early wilting and reduced tolerance of plants to drought stress (Virk et al., 2013). TRV-VIGS-mediated silencing of extracellular *PEROXIDASE 2* (*CaPO2*) in chili pepper resulted in increased susceptibility of silenced plants to mannitol-induced osmotic stress. Leaf disks from *CaPO2*-silenced leaves showed severe bleaching and higher chlorophyll loss than vector control plants (Choi and Hwang, 2012). Similarly, silencing of the *ABI3/VP1* transcription factor (*CaRAV1*) alone or together with *OXIDOREDUCTASE* (*CaOXR1*), using the TRV-VIGS vector, conferred reduced tolerance to mannitol-induced osmotic stress compared to vector control plants (Lee et al., 2010). This was accompanied by reduced expression of the known drought-stress-responsive genes *ANTIMICROBIAL PROTEIN* (*CaAMP1*) and *OSMOTIN* (*CaOSM1*) (Hong et al., 2004; Lee and Hwang, 2009). A recent study (Lim and Lee, 2014) implicated the involvement of *MILDEW RESISTANCE LOCUS O* (*CaMLO2*) in drought tolerance in chili pepper. Silencing of *CaMLO2* using the TRV-VIGS vector in chili pepper plants showed lower levels of transpirational water loss and lipid peroxidation in dehydrated leaves compared to wild-type plants. This study showed that *CaMLO2* acts as a negative regulator under drought stress conditions.

Another study demonstrated the usefulness of the TRV-based VIGS technique to study dehydration-responsive genes in rose flowers. Individual silencing of the *NAC TRANSCRIPTION FACTOR 2* (*RhNAC2*) and *A-TYPE EXPANSIN 4* (*RhEXPA4*) in rose petals and petal disks reduced the recovery of petals and petal disks during rehydration (Dai et al., 2012). Similarly, silencing of *NAC TRANSCRIPTION FACTOR 3* (*RhNAC3*) in rose petals has resulted in a decrease in cell expansion of the petals during rehydration along with concomitant down-regulation of several stress- and cell-expansion-related genes in the silenced petals compared to the vector control (Jiang et al., 2014). These genes are possible candidates for improving the shelf life of rose flowers through reduced water loss. Silencing of the *ACC SYNTHASE 1* (*RhACS1*) and *ACC SYNTHASE 2* (*RhACS2*) genes individually or co-silencing of both genes suppressed dehydration- and rehydration-induced ethylene in the sepals and gynoecia. Reduced ethylene production resulted in improved petal cell expansion during dehydration. On the contrary, silencing of an ethylene receptor, *RhETR3*, enhanced the inhibitory effect of dehydration on petal cell expansion (Liu et al., 2013). These results suggest that ethylene mediates dehydration-induced inhibition of cell expansion in rose petals.

VIGS has also been used to study drought stress response in monocotyledonous crop species. In a recent study (Manmathan et al., 2013), two drought-stress-responsive genes, *ENHANCED RESPONSE TO ABSCISIC ACID* (*Era1*) and *INOSITOL POLYPHOSPHATE 1-PHOSPHATASE* (*Sal1*), were individually silenced in wheat using the BSMV-VIGS vector. *Era1* gene encodes the β-subunit of farnesyltransferase involved in ABA mediated stomatal closure by activating the guard cell S-type anion-channels and increasing the cytosolic Ca^2+^ concentration. The loss-of-function of *Era1* has been shown to enhance ABA sensitivity and hence reduced stomatal conductance and water loss (Cutler et al., 1996; Allen et al., 2002; Wang et al., 2005). Similarly, *Sal1* has been shown to act as a negative regulator of both ABA-independent and ABA-dependent stress response pathways. Its loss-of-function has shown to increase the sensitivity of plants to drought stress (Wilson et al., 2009). *Era1*- and *Sal1*-silenced plants subjected to drought stress showed increased RWC, improved water use efficiency (WUE) and better vigor compared to vector-inoculated plants. This suggests that down-regulation of *Era1* and *Sal1* genes enhances drought tolerance in wheat by decreasing sensitivity to ABA. In another study, *H. VULGARIS ABUNDANT PROTEIN* (*HvHVA1*) and *DEHYDRIN 6* (*HvDhn6*), genes encoding the LEA class of proteins, were individually silenced in wheat using the BSMV-based VIGS vector (Liang et al., 2012). Under drought stress, both *HVA1*- and *Dhn6*-silenced plants showed lower survival rates than vector control plants. In addition, *HVA1*-silenced plants showed a higher rate of water loss under drought stress compared to vector control plants. However, the silenced plants also showed reduced vegetative growth and lower biomass even under well-watered conditions. This suggested the involvement of *HvHVA1* and *HvDhn6* in growth and development apart from drought tolerance (Liang et al., 2012). BSMV-VIGS-mediated silencing of the *BASIC TRANSCRIPTION FACTOR 3* (*TaBTF3*) gene in wheat resulted in a decreased plant survival rate, less free proline content, less RWC and increased membrane leakage compared to vector control plants under drought stress (Kang et al., 2013). Similarly, BSMV-VIGS-mediated silencing of *AUTOPHAGY-RELATED 8* (*TdAtg8*) from *Triticum dicoccoides* (wild emmer wheat) resulted in reduced chlorophyll content and an increase in malondialdehyde (MDA) content in silenced plants under drought stress (Kuzuoglu-Ozturk et al., 2012). The increased levels of MDA indicate membrane damage due to lipid peroxidation mainly by the effect of reactive oxygen species (ROS) (Zhang and Kirkham, 1994).

Taken together, these studies demonstrate the versatility of VIGS in deciphering the role of drought-stress-responsive genes in both dicotyledonous and monocotyledonous plants. In addition, the application of VIGS in silencing drought-stress-related genes in flowers (Dai et al., 2012) signifies its efficacy in studying the reproductive-tissue-associated genes which are important during terminal drought stress. Furthermore, VIGS has the potential to identify negative regulators of drought stress response during the reproductive stage.

### Salt-stress tolerance

The utility of VIGS in investigating salt stress tolerance in crop plants has also been demonstrated. *SlGRX1* gene silencing in tomato by a satellite DNAmβ-based VIGS vector resulted in yellowing of leaves under salinity stress compared to vector control plants due to a reduction in chlorophyll content, suggesting the role of *GRX1* in salt tolerance (Guo et al., 2010). Further, the role of *CaRAV1* and *CaOXR1* has been studied by TRV-VIGS in chili pepper (Lee et al., 2010). Leaf disks from *CaRAV1*-only silenced and *CaRAV1*/*CaOXR1* co-silenced plants exposed to different concentrations of NaCl showed severe bleaching due to loss of chlorophyll compared to vector control plants. Similarly, TRV-VIGS-mediated silencing of *CaPO2* resulted in a reduction in chlorophyll content and higher lipid peroxidation, leading to increased susceptibility of silenced chili pepper plants to salt stress compared to vector control plants (Choi and Hwang, 2012). Consistently, ectopic expression of *CaPO2* in Arabidopsis conferred enhanced tolerance to high salt stress, suggesting the role of *CaPO2* in salinity tolerance (Choi and Hwang, 2012). Taken together, these studies demonstrate the usefulness of VIGS in functional analysis of genes involved in salinity tolerance in crop plants.

### Oxidative stress tolerance

ROS increases in plants challenged by drought, salinity, extreme temperatures, or high light stress (Pastori and Foyer, 2002); this in turn leads to oxidative stress (Apel and Hirt, 2004). VIGS has been used to study oxidative stress tolerance in the recent past. A few studies (Lee et al., 2010; Choi and Hwang, 2012) described earlier in this review that examined the role of chili pepper genes, like *CaRAV1, CaOXR1*, and *CaPO2*, have also described oxidative stress damage in the plants with these genes silenced. Silencing of *CaRAV1, CaOXR1*, or *CaPO2* individually, or co-silencing of *CaRAV1*/*CaOXR1* in chili pepper resulted in enhanced lipid peroxidation under stress (Lee et al., 2010; Choi and Hwang, 2012). Similarly, downregulation of *CaMLO2* expression in chili pepper using TRV-based VIGS resulted in lower MDA levels under drought stress compared to vector control plants (Lim and Lee, 2014). This indicated the plausible negative role of *CaMLO2* under drought as well as oxidative stress. In wheat, silencing of *TdAtg8* using BSMV-based VIGS resulted in higher MDA levels compared to vector control under drought stress, thus suggesting the possible involvement of *TdAtg8* under oxidative stress (Kuzuoglu-Ozturk et al., 2012). High light stress induces oxidative stress in chloroplast. A recent study (Yuan-Ge et al., 2014) used BSMV-based VIGS to silence the *PROTON GRADIENT REGULATION 5* (*TaPGR5*) gene in wheat to test its involvement in tolerance to photo-inhibition under high light treatment. High light inhibited the net photosynthesis and affected the maximal quantum yield of Photosystem II (Fv/Fm) in the silenced plants. Also, silenced plants showed increased membrane damage, anthocyanin accumulation and higher MDA, suggesting the role of *TaPGR5* in oxidative stress tolerance. In pea, PEBV-VIGS-mediated co-silencing of thioredoxin genes, *TRX-F/TRX-M*, resulted in a significant reduction in Mg-chelatase activity and 5-aminolevulinic acid synthesizing capacity. This was associated with reduced chlorophyll and carotenoid pigment contents, lowered photosynthetic capacity and reduced expression of tetrapyrrole biosynthesis pathway genes, leading to the accumulation of ROS (Luo et al., 2012). Altogether, these studies highlight the utility of VIGS in characterizing the genes that mitigate oxidative stress in crop plants.

## VIGS for functional analysis of mineral nutrition-related genes in crop plants

Differential expression of a large number of genes in response to nutrient deficiency or toxicity has been shown in plants (Wang et al., 2002; Becher et al., 2004; Hirai et al., 2004; Takehisa et al., 2013), but only a few of them have been functionally characterized. In a soybean (*Glycine max*) iron-inefficient line, Isoclark, a *Bean pod mottle virus* (BPMV)-based VIGS vector was used to silence a *REPLICATION PROTEIN A* (*GmRPA3*) gene. *GmRPA3*-silenced plants had smaller leaves, decreased internode length and higher chlorophyll content, and failed to respond to increased iron nutrition, suggesting a role of the *GmRPA3* gene in iron acquisition (Atwood et al., 2014). Using a satellite DNA (DNAmβ) virus system with TYLCCNV, the function of *FERRIC CHELATE REDUCTASE* gene (*FRO1*) was studied in tomato roots (He et al., 2008). Silencing of *FRO1* resulted in reduced ferric chelate reductase activity in roots. In pea (*Pisum sativum*), a *Pea early browning virus* (PEBV)-based vector was used to study arbuscular-mycorrhizal-fungi (AMF)-associated phosphate acquisition. Silencing of a symbiotic gene, *PsSym19*, reduced the development of both arbuscules and vesicles at the root cortex. Similarly, silencing of a putative Pi transporter gene, *PsPT4*, using the PEBV-vector, reduced the phosphate uptake (Grønlund et al., 2010), suggesting the importance of these genes in phosphate assimilation in pea plants. Taken together, these studies suggest that VIGS can be effectively used to analyze gene function associated with nutrient deficiency in crop plants.

## Advantages of using VIGS to study abiotic stress tolerance in crop plants

VIGS has several advantages over most established functional genomics tools (Burch-Smith et al., 2004; Purkayastha and Dasgupta, 2009; Unver and Budak, 2009; Stratmann and Hind, 2011; Pflieger et al., 2013). (1) VIGS is faster and relatively easy to perform. VIGS can produce loss-of-function phenotype of a specific gene in a short period resulting in rapid functional characterization of genes (Dinesh-Kumar et al., 2003). (2) VIGS avoids plant transformation. Functional characterization of genes in difficult to transform species would be more easier once the VIGS system is established in that species (Burch-Smith et al., 2004). (3) VIGS allows functional analysis of genes whose loss-of-function produces lethal phenotype. It can be used to study genes related to embryonic development and seedling emergence and vigor (an important abiotic stress tolerance trait) (Ratcliff et al., 2001; Burch-Smith et al., 2004; Liu et al., 2004). (4) VIGS can overcome functional redundancy. Using the most conserved regions in VIGS, the multiple related genes or gene families can be silenced together (Ekengren et al., 2003; He et al., 2004). By silencing two or more members of the gene family with redundant functions the complex signaling components associated abiotic stresses such as drought can be deciphered. Though other functional genomics tools like antisense RNAs, artificial miRNAs, or RNAi can also be used for this purpose, but they are time consuming. (5) VIGS enables timely silencing of tissue-specific genes. For example, plants being infected only at the time of flowering or panicle development will predominantly have genes silenced in that organ. Besides, VIGS can be used to quickly silence genes in a particular gene mutant, stable RNAi or gene-overexpression plants. This will enable studying gene interactions under complex abiotic stresses in a large-scale and shorter time. In addition, VIGS is a feasible functional genomics tool over other PTGS-mediated gene silencing methods (Supplementary Table 3). VIGS is versatile, which allows rapid comparisons of gene function between species and works in different genetic backgrounds where genetic transformation is tedious and time consuming. VIGS also serves as a high-throughput forward as well as reverse genetics tool in plants. VIGS as a high-throughput reverse genetics tool can be performed by individually cloning fragments (usually 300–500 base pairs) from a large number of target genes into a suitable viral vector. The viral vector is delivered into plants using different methods. Abiotic stress can be applied 2–3 weeks after inoculation and the loss-of-function phenotype can be studied in the silenced plants to attribute function for the target gene under abiotic stress (Supplementary Figure 3). Similarly, VIGS as a forward genetics tool enables identification of critical players in stress tolerance. The stress specific cDNA pool can be cloned into binary vectors and transformed into *A. tumefaciens* in a high-throughput manner (Liu et al., 2002b). Each Agro-clone is inoculated into individual plants using a feasible inoculation method. The Agro-clones which produce interesting phenotype under abiotic stress can be quickly identified and sequenced to identify the inserted gene (Supplementary Figure 4). In addition to several general advantages, VIGS has some advantages pertinent to characterizing abiotic-stress-responsive genes.

## Limitations of VIGS in studying abiotic stress tolerance mechanisms and solutions to overcome the limitations

Though VIGS has been proved to be a robust tool for functional genomics studies, it has several limitations. These limitations and ways to overcome the same are listed below. (1) The virus vector may accumulate to high levels in the silenced plant if the silenced target gene is involved in the immunity of plants against the virus and such plants can become highly susceptible to subsequent abiotic stress. This will adversely influence studying the specific effect of gene silencing on abiotic stress tolerance. Quantification of viral load (Senthil-Kumar and Mysore, 2011b) in the silenced plants helps to decide whether the virus has accumulated higher than in the non-silenced control plant and this information can be used to choose different region of the target gene for silencing. (2) Virus infection by itself can interfere with abiotic stress response. For example, infection of *Brome mosaic virus* (BMV), *Cucumber mosaic virus* (CMV), *Tobacco mosaic virus* (TMV) and TRV delayed the appearance of drought symptoms in various plant species (Xu et al., 2008). The VIGS vector along with abiotic stress can create a scenario like concurrent biotic and abiotic stress. The phenotype produced under this situation might be different from abiotic stress alone (Suzuki et al., 2014). This can be overcome by including appropriate non-silenced vector control plants and comparing the results with specific gene silenced plants. (3) Silencing can be affected by changes in environmental conditions during abiotic stress treatment. Temperature, relative humidity and light can influence silencing (Fu et al., 2005, 2006; Kotakis et al., 2010). VIGS efficiency is reduced under high temperatures due to reduced virus multiplication (Chellappan et al., 2005). This can be overcome by verifying the viral multiplication beforehand and maintaining the VIGS vector-inoculated plants under optimum environmental conditions until the silencing followed by abiotic stress imposition. Ways to overcome some of the limitations of VIGS to study abiotic-stress-associated genes are also described in our earlier review (Senthil-Kumar and Udayakumar, 2010).

## Conclusion and future prospects

**Figure 1:**
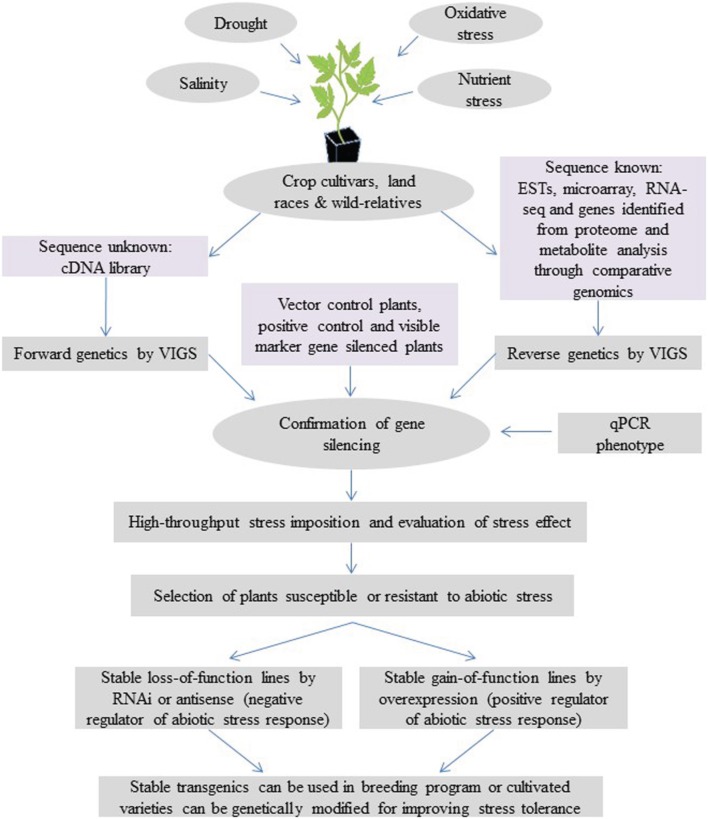
**Model showing the application of VIGS in understanding the mechanisms of abiotic stress tolerance and crop improvement**. VIGS can be used as a powerful reverse genetic tool for functional analysis of abiotic-stress-responsive genes identified from cultivars, land races and their wild relatives though transcriptome analysis and comparative analysis of molecular marker, proteome and metabolite data. VIGS can also be used for a high-throughput forward genetics screening. This is achieved by cloning the cDNA libraries generated from abiotic-stressed plants directly into a VIGS vector, inoculating them on target plants and analyzing the knockdown plants under abiotic stress. Along with target-gene-silenced plants, vector control and visible marker gene (like *phytoene desaturase, PDS* or *magnesium protoporphyrin chelatase subunit H, ChlH*)-silenced plants showing a photo-bleaching/yellowing phenotype will aid in identifying the time of initiation and duration of gene silencing. Silencing of a gene known to be involved in the specific abiotic stress tolerance that leads to susceptibility under stress (positive controls) is useful for coinciding abiotic stress imposition at the time of target gene silencing. In addition, high-throughput stress imposition and stress effect quantification methods can be used to screen large numbers of gene-silenced plants (Ramegowda et al., 2013). Candidate genes identified from the screen can be further confirmed by generating stable RNAi or overexpression transgenic lines. The trait can then be transferred to elite cultivars through breeding or generating transgenics in amenable cultivars to develop stress-tolerant crop plants.

VIGS, as both a forward and reverse genetics tool, offers opportunities for rapid functional analysis of abiotic-stress-related genes in both dicotyledonous and monocotyledonous crop species. Utilization of VIGS for understanding the mechanisms of abiotic stress tolerance and crop improvement is depicted in Figure 1. Currently, nearly 50 plant species have been shown to be amenable for VIGS (Lange et al., 2013), and VIGS is expected to be expanded to many other crop plants in future. Stress imposition protocols for VIGS plants have been optimized for several abiotic stresses, including drought, salinity and oxidative stress, and extreme temperatures (Ramegowda et al., 2013). Recently, a modified virus vector has been developed to express artificial and endogenous miRNAs in plants (Tang et al., 2010). Virus-vector-mediated silencing using artificial miRNA will be useful for functional analysis of abiotic-stress-associated miRNAs in crop plants. This approach will combine the specificity of amiRNA and versatility of VIGS. VIGS could also assist plant breeding programs in validating quantitative trait loci (QTL) and genes associated with abiotic stress traits (Cheng et al., 2010). Most of the QTL identified by molecular marker technologies would have multiple candidate genes. VIGS could serve as an effective and robust functional genomics tool to validate each gene in the locus. For example, a combination of cDNA-amplified fragment length polymorphism (AFLP) and VIGS can be used to screen a large number of genes and identify genes associated with abiotic stress tolerance. In summary, VIGS can play a major role in understanding abiotic stress tolerance mechanisms. This will have a direct impact on developing crop varieties that are tolerant to abiotic stress.

## Author contributions

Venkategowda Ramegowda and Muthappa Senthil-Kumar wrote the manuscript, and Kirankumar S. Mysore edited the manuscript.

### Conflict of interest statement

The authors declare that the research was conducted in the absence of any commercial or financial relationships that could be construed as a potential conflict of interest.
