# Supplementary material for: Virus-induced gene silencing is a versatile tool for unraveling the functional relevance of multiple abiotic-stress-responsive genes in crop plants
Source: Front Plant Sci. 2014 Jul 8;5:323. doi: 10.3389/fpls.2014.00323 (PMC4085877; doi:10.3389/fpls.2014.00323)
Supplement: Supplementary file 1 [file Presentation1.ZIP › fpls.2014.00323 - Presentation/86469__Presentation_1(1).ZIP!/Presentation 1/Supp. Mat. legends.docx]

**Tables**

**Supplementary Table 1.** VIGS vectors available for silencing genes in crop plants.

**Supplementary Table 2:** Different methods of delivering VIGS vector into plants.

**Supplementary Table 3:** Advantages of VIGS over other PTGS-mediated gene silencing methods in plants.

**Figure Legend**

**Supplementary Figure 1: Molecular mechanism of virus-induced gene silencing (VIGS) in plants.** Delivery of recombinant viral vector carrying viral genes along with target plant gene fragment results in production of long double stranded RNA (dsRNA) by RNA-dependent RNA polymerase (RdRp) (Dalmay et al., 2000; Mourrain et al., 2000). The central part of VIGS-mediated gene silencing is the generation of short interfering RNAs (siRNAs) of 21-25- nucleotide from dsRNA by RNAse, DICER-LIKE proteins (DCL2/DCL4) (Deleris et al., 2006) in interaction with DSRNA BINDING PROTEIN (DRB) (Brodersen and Voinnet 2006). Such siRNA duplexes may undergo HUA ENHANCER protein (HEN1)-mediated methylation at their 3′ end (Boutet et al., 2003). The siRNAs are then incorporated into the RNA-induced silencing complex (RISC) and guide cleavage of target RNAs by ARGONAUTE protein (AGO1) (Fagard et al. 2000; Morel et al., 2002). The cleaved RNA can be further used as a source of dsRNA production. Orange and green colored lines indicate viral genome (+ and – strands); Blue and pink colored lines indicate plant gene (+ and – strands); Green oval indicates viral or plant origin; other ovals indicate plant origin.

**Supplementary Figure 2: Duration of gene silencing in the VIGS plants and transfer to progeny.** (a) Short duration VIGS: In general, VIGS is known to occur for a shorter duration of the life cycle of a plant, for about 3 weeks, and is termed as short duration VIGS (Ryu et al., 2004). The efficiency would decrease after a month and plants would start recovering from the silencing (Ratcliff et al., 2001). Short duration VIGS can be achieved by inoculating 3-4 leaf stage plants with recombinant VIGS vector using different VIGS vector delivery methods. Efficient silencing occurs at 2-3 weeks after inoculation and at this stage plants can be used to analyze the silencing effect. (b) Long duration VIGS: Efficient silencing can continue for months some times through the entire life cycle of the plant which can be referred to as long duration VIGS (Senthil-Kumar and Mysore 2011). Agrodrench coupled with leaf inoculation has been suggested to induce effective silencing for a longer duration. Viral titer may decrease after 3-4 months and hence booster dose inoculation of recombinant virus every 3 months will be useful to maintain silencing for a long duration. The effectiveness of long duration silencing has been shown to be influenced by the inoculation method, viral titer and maintenance of environmental conditions for healthy plant growth. (c) Transmittance of VIGS to progeny plants: VIGS can occur for years and also be transmitted to progeny plants. BSMV-, ASLV and TRV-mediated VIGS has been shown to be transmitted to progeny plants with different efficiencies (Rodrigo et al., 2011; Pflieger et al., 2008; Senthil-Kumar and Mysore 2011). Here, booster dose inoculation every 3 months and maintenance of environmental conditions suitable for viral multiplication and new plant growth throughout the life cycle of the plant is important. ^*^months are only examples, depending on the crop the duration varies; ^**^booster dose is not required when VIGS persists and required only when plants start recovering.

**Supplementary Figure 3. Methodology of VIGS and reverse genetics-based functional characterization of genes using VIGS.** To use VIGS as reverse genetics tool, the cDNA of candidate gene is selected, and potential region that can generate efﬁcient siRNAs and has no sequence similarity to any other off-target genes is identified using siRNA scan software (<http://bioinfo2.noble.org/RNAiScan.htm>) (Xu et al., 2006). A fragment of (usually 300-500-bp) is cloned into a viral genome under the control the CaMV35S promoter in a binary vector. The recombinant viral vector is delivered into plants using different methods. The most widely used method is transformation of recombinant viral vector into *Agrobacterium tumefaciens* and inoculating plants by a toothpick, vacuum infiltration, syringe infiltration or Agrodrench (Burch-Smith et al., 2004; Senthil-Kumar and Mysore, 2014). The other methods of viral vector delivery are, the use of intermediate host for Agro-inoculation and rubbing the sap extracted from intermediate host onto target plant; direct DNA deliver either through biolistic method or rubbing the plasmid on to leaves; and rubbing the *in vitro* transcribed RNA onto leaves (Nagamatsu et al., 2007; Fofana et al., 2004; Zhang et al., 2010). The virus spreads systemically from the site of delivery to newly growing plant parts and triggers plant defense mechanism resulting in degradation of both viral RNA and endogenous RNA of the target gene in the host (Liu et al., 2002). After 2-3 weeks, the plants can be used for analysis.

**Supplementary Figure 4. High-throughput functional analysis of plant genes using VIGS as forward genetics tool.** Improvements in viral vectors and inoculation methods have enabled the use of VIGS for high-throughput screening of a large number of genes in a shorter period (Senthil-Kumar et al., 2013). For a forward genetics screening, cDNA pool generated from any experiments can be cloned into a Gateway enabled VIGS vector and transformed into *Agrobacterium tumefaciens* and stored in 96 well plates (Liu et al., 2002). Each Agro-clone is inoculated into individual plants using toothpick, a feasible inoculation method (Senthil-Kumar et al., 2013). Silencing will occur after 2-3 weeks of inoculation in the new growth and the abiotic stress phenotypes can be observed during that time. The plants which produce interesting phenotype can be quickly identified and the genes responsible for can be easily known by sequencing the insert in the corresponding VIGS construct.
