# Supplementary material for: Virus-induced gene silencing is a versatile tool for unraveling the functional relevance of multiple abiotic-stress-responsive genes in crop plants
Source: Front Plant Sci. 2014 Jul 8;5:323. doi: 10.3389/fpls.2014.00323 (PMC4085877; doi:10.3389/fpls.2014.00323)
Supplement: Supplementary file 1 [file Presentation1.ZIP › fpls.2014.00323 - Presentation/86469__Presentation_1(1).ZIP!/Presentation 1/Supplementary Tables 1-3.DOCX]

**Supplementary Table 1.** VIGS vectors available for silencing genes in crop plants

| **Sl. No.** | **Viruses** | **Virus type/ Group** | **Natural host species (examples)** | **Target crop species amenable for VIGS** | **Reference** |
| --- | --- | --- | --- | --- | --- |
| 1. | *Apple latent spherical virus* (ALSV) | RNA/ Cheravirus | *M. domestica* | *Solanum lycopersicum, Glycine max, Pisum sativum, Vigna angularis, V. unguiculata, Cucumis sativus, Cucurbita pepo, Citrullus lanatus, Luffa cylindrica, Lagenaria siceraria, Malus domestica, Pyrus sps.* | (Igarashi et al., 2009;Yamagishi and Yoshikawa, 2009;Sasaki et al., 2011) |
| 2. | *Barley stripe mosaic virus* (BSMV) | RNA/ Hordeivirus | *H. vulgare, T. aestivum A. sativa, Z. mays, Spinacia oleracea* | *Hordeum vulgare, Triticum aestivum, Avena sativa, Zingiber officinale* | (Holzberg et al., 2002;Bruun-Rasmussen et al., 2007;Renner et al., 2009;Pacak et al., 2010;Van Eck et al., 2010;Tufan et al., 2011;Yuan et al., 2011;Lee et al., 2012;Deng et al., 2013;Kang et al., 2013;Manmathan et al., 2013) |
| 3. | *Bean pod mottle virus (*BPMV) | RNA/ Comovirus | *P. vulgaris, G. max* | *G. max, Phaseolus vulgari* | (Zhang and Ghabrial, 2006;Zhang et al., 2009;Zhang et al., 2010;Juvale et al., 2012;Morales et al., 2013) |
| 4. | *Brome mosaic virus* (BMV) | RNA/ Bromovirus | *H. vulgare* | *H. vulgare, Oryza sativa, Z. Mays, Festuca arundinacea* | (Ding et al., 2006;van der Linde et al., 2011;Benavente et al., 2012) |
| 5. | *Cucumber mosaic virus* (CMV) | RNA/ Cucumovirus | *Cucurbita sps., S. lycopersicon, S. oleracea* | *G. max, Antirrhinum majus* | (Nagamatsu et al., 2007;Kim et al., 2011) |
| 6. | *Cymbidium mosaic virus* (CymMV) | RNA/ *Potexvirus* | *Orchidaceae genus* | *Phalaenopsis sps.* | (Lu et al., 2007) |
| 7. | *Grapevine leaf roll-associated virus-2* (GLRaV2) | RNA/ Closterovirus | *V. vinifera* | *V. vinifera* | (Kurth et al., 2012) |
| 8. | *Pea early browning virus* (PEBV) | RNA/ Tobravirus | *P. sativum, P. vulgaris* | *P. sativum* | (Constantin et al., 2004;Constantin et al., 2008;Grønlund et al., 2010;Luo et al., 2012;Luo et al., 2013;Chen et al., 2014) |
| 9. | *Potato virus X* (PVX) | RNA/ *Potexvirus* |  | *Solanum tuberosum* | (Faivre-Rampant et al., 2004) |
| 10. | *Tobacco rattle virus* (TRV) | RNA/ Tobravirus | *S. oleracea, Beta vulgaris, S. tuberosum, Nicotiana tabacum* | *S. lycopersicum, Capsicum annuum, S. tuberosum, Gossypium hirsutum, Gerbera hybrida, Fragaria ananassa, Solanum melongena, Vaccinium myrtillus, Catharanthus roseus, Rosa hybrid* | (Liu et al., 2002;Brigneti et al., 2004;Chung et al., 2004;Orzaez et al., 2009;Jaakola et al., 2010;Wangdi et al., 2010;Liscombe and O'Connor, 2011;Dai et al., 2012;Liu et al., 2012;Qu et al., 2012;Jia and Shen, 2013;Wang et al., 2013;Jiang et al., 2014) |
| 11. | *White clover mosaic virus* (WClMV) | RNA/ Potexvirus | *Trifolium spp, P. sativum, Vicia faba, P. vulgaris, V. unguiculata, C. sativus, C. pepo, S. lycopersicum* | *P. sativum* | (Ido et al., 2012) |
| 12. | *African cassava mosaic virus* (ACMV) | DNA/ Begomovirus | *M. esculenta* | *Manihot esculenta* | (Fofana et al., 2004) |
| 13. | *Beet curly top virus* (BCTV) | DNA/ Curtovirus | *B. vulgaris, C. annuum, S.lycopersicum* | *S. olerace, S. lycopersicum* | (Golenberg et al., 2009) |
| 14. | *Cotton leaf crumple*  *Virus* (CLCrV) | DNA/ *Begomovirus* | *G. hirsutum, Malva parviflora, Hibiscus cannabinus, P. vulgaris* | *G. hirsutum* | (Tuttle et al., 2008;Idris et al., 2010) |
| 15. | *Grapevine virus A* (GVA) | DNA/ *Vitivirus* | *Vitis coignetiae,*  *V. vinifera* | *Vitis vinifera* | (Muruganantham et al., 2009) |
| 16. | *Pepper huasteco yellow vein virus* (PHYVV) | DNA/ Begomovirus | *C. annuum, N. Tabacum, N. benthamiana* | *C. annuum* | (Rosario Abraham-Juárez et al., 2008) |
| 17. | *Rice tungro bacilliform*  *Virus* (RTBV) | DNA/ Tungrovirus | *O. sativa* | *O. sativa* | (Purkayastha et al., 2010) |
| 18. | *Tobacco curly shoot virus* (TbCSV) *Alphasatellite* | DNA/ Alphasatellite DNA | *N. tabacum, S. lycopersicum, Cucurbita moschata* | *S. lycopersicum* | (Huang et al., 2009) |
| 19. | *Tomato leaf curl virus*  (ToLCV) | DNA/ *Begomovirus* | *S. lycopersicum* | *S. lycopersicum* | (Pandey et al., 2009) |
| 20. | *Tomato yellow leaf curl China Virus* (TYLCCNV) | DNA/ DNAbeta satellite DNA | *S. lycopersicum* | *S. lycopersicum* | (Tao and Zhou, 2004) |
| 21. | *Euphorbia mosaic virus-Yucatan peninsula* (EuMV-YP) | DNA/ Begomovirus | *P. vulgaris, C. Annuum, S. Lycopersicum, Datura stramonium* | *C. annuum* | (Villanueva-Alonzo et al., 2013) |
| 22. | *Grapevine leaf roll-associated virus-2* (GLRaV2) | RNA/ Closterovirus | *V. vinifera* | *V. vinifera* | (Kurth et al., 2012) |

**Supplementary Table 2:** Different methods of delivering VIGS vector into plants.

| **Sl. No** | **Method of delivery of VIGS vector into plants** | **VIGS vector** | **Reference** |
| --- | --- | --- | --- |
| 1 | **Agro-inoculation:**  Transformation of *A. tumefaciens* with the recombinant viral vector plasmid and Agro-inoculation of leaves by toothpick, vacuum infiltration or Agrodrench | PVX; PEBV; TRV; ACMV; BCTV; CLCrV; GVA; RTBV; ToLCV; TYLCCNV; TbCSV; BMV | (Ratcliff et al., 2001;Faivre-Rampant et al., 2004;Fofana et al., 2004;Tao and Zhou, 2004;Ding et al., 2006;Constantin et al., 2008;Tuttle et al., 2008;Golenberg et al., 2009;Huang et al., 2009;Muruganantham et al., 2009;Pandey et al., 2009;Purkayastha et al., 2010) |
| 2 | **Rubbing of RNA transcripts:**  *In vitro* transcription and mechanical rub-inoculation of RNA | BMV; BSMV; CMV; CymMV | (Holzberg et al., 2002;Ding et al., 2006;Lu et al., 2007;Nagamatsu et al., 2007) |
| 3 | **Biolistic:**  Bombardment of leaves with gold particle coated viral vector plasmids using gene gun | BPMV; ACMV; BCTV; CLCrV; PHYVV | (Fofana et al., 2004;Rosario Abraham-Juárez et al., 2008;Tuttle et al., 2008;Golenberg et al., 2009;Zhang et al., 2009) |
| 4 | **Direct DNA rubbing:**  Mechanical rub-inoculation of viral vector plasmids | BPMV | (Zhang et al., 2010) |
| 5 | **Rubbing of sap from intermediate host:**  Agro-inoculation of intermediate host and rub-inoculation of sap from intermediate host (for example, *Nicotiana benthamiana*) | ALSV; CMV | (Nagamatsu et al., 2007;Igarashi et al., 2009) |

**Supplementary Table 3:** Advantages of VIGS over other PTGS-mediated gene silencing methods in plants.

| **Description** | **VIGS** | **RNAi** | **Antisense** | **amiRNA** |
| --- | --- | --- | --- | --- |
| Genetic transformation | Not required | Required | Required | Required |
| Duration | Short (1 – 2 months) | More than 6 months because of genetic transformation; depends on plant species | More than 6 months because of genetic transformation; depends on plant species | More than 6 months because of genetic transformation; depends on plant species |
| Ease and Cost | Easy and low cost | Difficult for several crop plants and expensive | Difficult for several crop plants and expensive | Difficult for several crop plants and expensive |
| High throughput screening | Fast-forward and reverse genetics possible | Not feasible | Not feasible | Not feasible |
| Efficiency of silencing | Highly efficient; depends on the virus-host specificity | Highly efficient | Highly efficient | Quantitative silencing, depends on the promoter strength |
| Rapid comparisons of gene function between species | Possible | No | No | No |
| Working in different genetic backgrounds | Continuous improvements in vectors and inoculation methods has enabled the use of VIGS in more than 50 plant species | Possible in species where genetic transformation has been established | Possible in species where genetic transformation has been established | Possible in species where genetic transformation has been established |
| Tissue specificity | Possible | Possible with the use of tissue specific promoter | Possible with the use of tissue specific promoter | Possible with the use of tissue specific promoter |

**References**

Benavente, L.M., Ding, X.S., Redinbaugh, M.G., Nelson, R.S., and Balint-Kurti, P. (2012). Virus-induced gene silencing in diverse maize lines using the *Brome mosaic virus*-based silencing vector. *Maydica* 57**,** 206-214.

Brigneti, G., Martín-Hernández, A.M., Jin, H., Chen, J., Baulcombe, D.C., Baker, B., and Jones, J.D. (2004). Virus-induced gene silencing in *Solanum species*. *Plant J* 39**,** 264-272.

Bruun-Rasmussen, M., Madsen, C.T., Jessing, S., and Albrechtsen, M. (2007). Stability of *Barley stripe mosaic virus*-induced gene silencing in barley. *Mol Plant Microbe Interact* 20**,** 1323-1331.

Chen, Z.H., Jia, F.F., Hu, J.Q., Pang, J.L., Xu, L., and Wang, L.L. (2014). Virus-induced gene silencing of PEAM4 affects floral morphology by altering the expression pattern of PsSOC1a and PsPVP in pea. *J Plant Physiol* 171**,** 148-153.

Chung, E., Seong, E., Kim, Y.C., Chung, E.J., Oh, S.K., Lee, S., Park, J.M., Joung, Y.H., and Choi, D. (2004). A method of high frequency virus-induced gene silencing in chili pepper (*Capsicum annuum* L. cv. *Bukang*). *Mol Cells* 17**,** 377-380.

Constantin, G.D., Grønlund, M., Johansen, I.E., Stougaard, J., and Lund, O.S. (2008). Virus-induced gene silencing (VIGS) as a reverse genetic tool to study development of symbiotic root nodules. *Mol Plant Microbe Interact* 21**,** 720-727.

Constantin, G.D., Krath, B.N., Macfarlane, S.A., Nicolaisen, M., Johansen, I.E., and Lund, O.S. (2004). Virus-induced gene silencing as a tool for functional genomics in a legume species. *Plant J* 40**,** 622-631.

Dai, F., Zhang, C., Jiang, X., Kang, M., Yin, X., Lü, P., Zhang, X., Zheng, Y., and Gao, J. (2012). RhNAC2 and RhEXPA4 are involved in the regulation of dehydration tolerance during the expansion of rose petals. *Plant Physiol* 160**,** 2064-2082.

Deng, X., Kelloniemi, J., Haikonen, T., Vuorinen, A.L., Elomaa, P., Teeri, T.H., and Valkonen, J.P. (2013). Modification of *Tobacco rattle virus* RNA1 to serve as a VIGS vector reveals that the 29K movement protein is an RNA silencing suppressor of the virus. *Mol Plant Microbe Interact* 26**,** 503-514.

Ding, X.S., Schneider, W.L., Chaluvadi, S.R., Mian, M.A., and Nelson, R.S. (2006). Characterization of a *Brome mosaic virus* strain and its use as a vector for gene silencing in monocotyledonous hosts. *Mol Plant Microbe Interact* 19**,** 1229-1239.

Faivre-Rampant, O., Gilroy, E.M., Hrubikova, K., Hein, I., Millam, S., Loake, G.J., Birch, P., Taylor, M., and Lacomme, C. (2004). *Potato virus X*-induced gene silencing in leaves and tubers of potato. *Plant Physiol* 134**,** 1308-1316.

Fofana, I.B., Sangaré, A., Collier, R., Taylor, C., and Fauquet, C.M. (2004). A geminivirus-induced gene silencing system for gene function validation in cassava. *Plant Mol Biol* 56**,** 613-624.

Golenberg, E.M., Sather, D.N., Hancock, L.C., Buckley, K.J., Villafranco, N.M., and Bisaro, D.M. (2009). Development of a gene silencing DNA vector derived from a broad host range geminivirus. *Plant Methods* 5**,** 9.

Grønlund, M., Olsen, A., Johansen, E.I., and Jakobsen, I. (2010). Protocol: using virus-induced gene silencing to study the arbuscular mycorrhizal symbiosis in *Pisum sativum*. *Plant Methods* 6**,** 28.

Holzberg, S., Brosio, P., Gross, C., and Pogue, G.P. (2002). *Barley stripe mosaic virus*-induced gene silencing in a monocot plant. *Plant J* 30**,** 315-327.

Huang, C., Xie, Y., and Zhou, X. (2009). Efficient virus-induced gene silencing in plants using a modified geminivirus DNA1 component. *Plant Biotechnol J* 7**,** 254-265.

Ido, Y., Nakahara, K., and Uyeda, I. (2012). *White clover mosaic virus*-induced gene silencing in pea. *J Gen Plant Pathol* 78**,** 127-132.

Idris, A.M., Tuttle, J.R., Robertson, D., Haigler, C.H., and Brown, J.K. (2010). Differential Cotton leaf crumple virus-VIGS-mediated gene silencing and viral genome localization in different *Gossypium hirsutum* genetic backgrounds. *Physiol Mol Plant P* 75**,** 13-22.

Igarashi, A., Yamagata, K., Sugai, T., Takahashi, Y., Sugawara, E., Tamura, A., Yaegashi, H., Yamagishi, N., Takahashi, T., Isogai, M., Takahashi, H., and Yoshikawa, N. (2009). Apple latent spherical virus vectors for reliable and effective virus-induced gene silencing among a broad range of plants including tobacco, tomato, *Arabidopsis thaliana*, cucurbits, and legumes. *Virology* 386**,** 407-416.

Jaakola, L., Poole, M., Jones, M.O., Kämäräinen-Karppinen, T., Koskimäki, J.J., Hohtola, A., Häggman, H., Fraser, P.D., Manning, K., King, G.J., Thomson, H., and Seymour, G.B. (2010). A SQUAMOSA MADS box gene involved in the regulation of anthocyanin accumulation in bilberry fruits. *Plant Physiol* 153**,** 1619-1629.

Jia, H., and Shen, Y. (2013). Virus-induced gene silencing in strawberry fruit. *Methods Mol Biol* 975**,** 211-218.

Jiang, X., Zhang, C., Lü, P., Jiang, G., Liu, X., Dai, F., and Gao, J. (2014). RhNAC3, a stress-associated NAC transcription factor, has a role in dehydration tolerance through regulating osmotic stress-related genes in rose petals. *Plant Biotechnol J* 12**,** 38-48.

Juvale, P.S., Hewezi, T., Zhang, C., Kandoth, P.K., Mitchum, M.G., Hill, J.H., Whitham, S.A., and Baum, T.J. (2012). Temporal and spatial *Bean pod mottle virus*-induced gene silencing in soybean. *Mol Plant Pathol* 13**,** 1140-1148.

Kang, G., Li, G., Ma, H., Wang, C., and Guo, T. (2013). Proteomic analysis on the leaves of TaBTF3 gene virus-induced silenced wheat plants may reveal its regulatory mechanism. *J Proteomics* 83**,** 130-143.

Kim, B., Inaba, J.-I., and Masuta, C. (2011). Virus induced gene silencing in *Antirrhinum majus* using the *Cucumber mosaic virus* vector: Functional analysis of the AINTEGUMENTA (Am-ANT) gene of *A. majus*. *Hortic Environ Biotechnol* 52**,** 176-182.

Kurth, E.G., Peremyslov, V.V., Prokhnevsky, A.I., Kasschau, K.D., Miller, M., Carrington, J.C., and Dolja, V.V. (2012). Virus-derived gene expression and RNA interference vector for grapevine. *J Virol* 86**,** 6002-6009.

Lee, W.S., Hammond-Kosack, K.E., and Kanyuka, K. (2012). *Barley stripe mosaic virus*-mediated tools for investigating gene function in cereal plants and their pathogens: virus-induced gene silencing, host-mediated gene silencing, and virus-mediated overexpression of heterologous protein. *Plant Physiol* 160**,** 582-590.

Liscombe, D.K., and O'connor, S.E. (2011). A virus-induced gene silencing approach to understanding alkaloid metabolism in *Catharanthus roseus*. *Phytochemistry* 72**,** 1969-1977.

Liu, H., Fu, D., Zhu, B., Yan, H., Shen, X., Zuo, J., Zhu, Y., and Luo, Y. (2012). Virus-induced gene silencing in eggplant (*Solanum melongena*). *J Integr Plant Biol* 54**,** 422-429.

Liu, Y., Schiff, M., and Dinesh-Kumar, S.P. (2002). Virus-induced gene silencing in tomato. *Plant J* 31**,** 777-786.

Lu, H.C., Chen, H.H., Tsai, W.C., Chen, W.H., Su, H.J., Chang, D.C., and Yeh, H.H. (2007). Strategies for functional validation of genes involved in reproductive stages of orchids. *Plant Physiol* 143**,** 558-569.

Luo, T., Fan, T., Liu, Y., Rothbart, M., Yu, J., Zhou, S., Grimm, B., and Luo, M. (2012). Thioredoxin redox regulates ATPase activity of magnesium chelatase CHLI subunit and modulates redox-mediated signaling in tetrapyrrole biosynthesis and homeostasis of reactive oxygen species in pea plants. *Plant Physiol* 159**,** 118-130.

Luo, T., Luo, S., Araújo, W.L., Schlicke, H., Rothbart, M., Yu, J., Fan, T., Fernie, A.R., Grimm, B., and Luo, M. (2013). Virus-induced gene silencing of pea CHLI and CHLD affects tetrapyrrole biosynthesis, chloroplast development and the primary metabolic network. *Plant Physiol Biochem* 65**,** 17-26.

Manmathan, H., Shaner, D., Snelling, J., Tisserat, N., and Lapitan, N. (2013). Virus-induced gene silencing of *Arabidopsis thaliana* gene homologues in wheat identifies genes conferring improved drought tolerance. *J Exp Bot* 64**,** 1381-1392.

Morales, A.M.A.P., O’rourke, J.A., Van De Mortel, M., Scheider, K.T., Bancroft, T.J., Borém, A., Nelson, R.T., Nettleton, D., Baum, T.J., Shoemaker, R.C., Frederick, R.D., Abdelnoor, R.V., Pedley, K.F., Whitham, S.A., and Graham, M.A. (2013). Transcriptome analyses and virus induced gene silencing identify genes in the Rpp4-mediated Asian soybean rust resistance pathway. *Funct Plant Biol* 40**,** 1029-1047.

Muruganantham, M., Moskovitz, Y., Haviv, S., Horesh, T., Fenigstein, A., Preez, J., Stephan, D., Burger, J.T., and Mawassi, M. (2009). *Grapevine virus A*-mediated gene silencing in *Nicotiana benthamiana* and *Vitis vinifera*. *J Virol Methods* 155**,** 167-174.

Nagamatsu, A., Masuta, C., Senda, M., Matsuura, H., Kasai, A., Hong, J.S., Kitamura, K., Abe, J., and Kanazawa, A. (2007). Functional analysis of soybean genes involved in flavonoid biosynthesis by virus-induced gene silencing. *Plant Biotechnol J* 5**,** 778-790.

Orzaez, D., Medina, A., Torre, S., Fernández-Moreno, J.P., Rambla, J.L., Fernández-Del-Carmen, A., Butelli, E., Martin, C., and Granell, A. (2009). A visual reporter system for virus-induced gene silencing in tomato fruit based on anthocyanin accumulation. *Plant Physiol* 150**,** 1122-1134.

Pacak, A., Geisler, K., Jørgensen, B., Barciszewska-Pacak, M., Nilsson, L., Nielsen, T.H., Johansen, E., Grønlund, M., Jakobsen, I., and Albrechtsen, M. (2010). Investigations of barley stripe mosaic virus as a gene silencing vector in barley roots and in *Brachypodium distachyon* and oat. *Plant Methods* 6**,** 26.

Pandey, P., Choudhury, N.R., and Mukherjee, S.K. (2009). A geminiviral amplicon (VA) derived from *Tomato leaf curl virus* (ToLCV) can replicate in a wide variety of plant species and also acts as a VIGS vector. *Virol J* 6**,** 152.

Purkayastha, A., Mathur, S., Verma, V., Sharma, S., and Dasgupta, I. (2010). Virus-induced gene silencing in rice using a vector derived from a DNA virus. *Planta* 232**,** 1531-1540.

Qu, J., Ye, J., Geng, Y.F., Sun, Y.W., Gao, S.Q., Zhang, B.P., Chen, W., and Chua, N.H. (2012). Dissecting functions of KATANIN and WRINKLED1 in cotton fiber development by virus-induced gene silencing. *Plant Physiol* 160**,** 738-748.

Ratcliff, F., Martin-Hernandez, A.M., and Baulcombe, D.C. (2001). Technical Advance. *Tobacco rattle virus* as a vector for analysis of gene function by silencing. *Plant J* 25**,** 237-245.

Renner, T., Bragg, J., Driscoll, H.E., Cho, J., Jackson, A.O., and Specht, C.D. (2009). Virus-induced gene silencing in the culinary ginger (*Zingiber officinale*): an effective mechanism for down-regulating gene expression in tropical monocots. *Mol Plant* 2**,** 1084-1094.

Rosario Abraham-Juárez, M., Carmen Rocha-Granados, M., López, M., Rivera-Bustamante, R., and Ochoa-Alejo, N. (2008). Virus-induced silencing of Comt, pAmt and Kas genes results in a reduction of capsaicinoid accumulation in chili pepper fruits. *Planta* 227**,** 681-695.

Sasaki, S., Yamagishi, N., and Yoshikawa, N. (2011). Efficient virus-induced gene silencing in apple, pear and Japanese pear using *Apple latent spherical virus* vectors. *Plant Methods* 7**,** 15.

Tao, X., and Zhou, X. (2004). A modified viral satellite DNA that suppresses gene expression in plants. *Plant J* 38**,** 850-860.

Tufan, H.A., Stefanato, F.L., Mcgrann, G.R., Maccormack, R., and Boyd, L.A. (2011). The *Barley stripe mosaic virus* system used for virus-induced gene silencing in cereals differentially affects susceptibility to fungal pathogens in wheat. *J Plant Physiol* 168**,** 990-994.

Tuttle, J.R., Idris, A.M., Brown, J.K., Haigler, C.H., and Robertson, D. (2008). Geminivirus-mediated gene silencing from *Cotton leaf crumple virus* is enhanced by low temperature in cotton. *Plant Physiol* 148**,** 41-50.

Van Der Linde, K., Kastner, C., Kumlehn, J., Kahmann, R., and Doehlemann, G. (2011). Systemic virus-induced gene silencing allows functional characterization of maize genes during biotrophic interaction with *Ustilago maydis*. *New Phytol* 189**,** 471-483.

Van Eck, L., Schultz, T., Leach, J.E., Scofield, S.R., Peairs, F.B., Botha, A.M., and Lapitan, N.L. (2010). Virus-induced gene silencing of WRKY53 and an inducible phenylalanine ammonia-lyase in wheat reduces aphid resistance. *Plant Biotechnol J* 8**,** 1023-1032.

Villanueva-Alonzo, H.J., Us-Camas, R.Y., López-Ochoa, L.A., Robertson, D., Guerra-Peraza, O., Minero-García, Y., and Moreno-Valenzuela, O.A. (2013). A new virus-induced gene silencing vector based on *Euphorbia mosaic virus-Yucatan peninsula* for NPR1 silencing in *Nicotiana benthamiana* and *Capsicum annuum* var. *Anaheim*. *Biotechnol Lett* 35**,** 811-823.

Wang, J.E., Li, D.W., Gong, Z.H., and Zhang, Y.L. (2013). Optimization of virus-induced gene silencing in pepper (*Capsicum annuum* L.). *Genet Mol Res* 12**,** 2492-2506.

Wangdi, T., Uppalapati, S.R., Nagaraj, S., Ryu, C.M., Bender, C.L., and Mysore, K.S. (2010). A virus-induced gene silencing screen identifies a role for Thylakoid Formation1 in *Pseudomonas syringae* pv *tomato* symptom development in tomato and Arabidopsis. *Plant Physiol* 152**,** 281-292.

Yamagishi, N., and Yoshikawa, N. (2009). Virus-induced gene silencing in soybean seeds and the emergence stage of soybean plants with *Apple latent spherical virus* vectors. *Plant Mol Biol* 71**,** 15-24.

Yuan, C., Li, C., Yan, L., Jackson, A.O., Liu, Z., Han, C., Yu, J., and Li, D. (2011). A high throughput *Barley stripe mosaic virus* vector for virus induced gene silencing in monocots and dicots. *PLoS One* 6**,** e26468.

Zhang, C., Bradshaw, J.D., Whitham, S.A., and Hill, J.H. (2010). The development of an efficient multipurpose *Bean pod mottle virus* viral vector set for foreign gene expression and RNA silencing. *Plant Physiol* 153**,** 52-65.

Zhang, C., and Ghabrial, S.A. (2006). Development of *Bean pod mottle virus*-based vectors for stable protein expression and sequence-specific virus-induced gene silencing in soybean. *Virology* 344**,** 401-411.

Zhang, C., Yang, C., Whitham, S.A., and Hill, J.H. (2009). Development and use of an efficient DNA-based viral gene silencing vector for soybean. *Mol Plant Microbe Interact* 22**,** 123-131.
